# Analysis of Short-Term Efficacy of Gasless Single-Port Laparoscopic Inguinal Lymphadenectomy Through Vulva Incision for Vulvar Cancer

**DOI:** 10.3389/fsurg.2022.813711

**Published:** 2022-03-24

**Authors:** Jin Ding, Piaopiao Teng, Xiaoming Guan, Yonghong Luo, Huafeng Ding, Suhua Shi, Xiufen Zhou, Guantai Ni

**Affiliations:** ^1^Department of Obstetrics and Gynecology, First Affiliated Hospital of Wannan Medical College, Wuhu, China; ^2^Department of Obstetrics and Gynecology, Baylor College of Medicine, Houston, TX, United States; ^3^Department of Gynecology, Lu'an Affiliated Hospital of Anhui Medical University, Lu'an, China

**Keywords:** vulvar cancer, laparoscopy, inguinal lymphadenectomy, single-port, surgery

## Abstract

**Objective:**

To investigate the feasibility and short-term efficacy of gasless single-port laparoscopic inguinal lymphadenectomy through vulva incision (VEIL-V).

**Methods:**

The data of 9 patients diagnosed as vulvar squamous cell carcinoma who underwent single-port laparoscopic inguinal lymph node dissection through vulvectomy incision were retrospectively analyzed. And 13 patients who underwent laparoscopic inguinal lymph node dissection through lower abdominal subcutaneous approach as the control group (VEIL-H). The operation time, blood loss, numbers of unilateral lymph nodes, hospitalization time, and complications between the two groups were compared.

**Results:**

The operation time of VEIL-V was 56.11 ± 5.94 min, which were shorter than that of VEIL-H (74.62 ± 5.50 min; *P* = 0.013). Bleeding amount in the VEIL-H was 29.44 ± 2.56, which was significantly lower than that of the VEIL-H group (43.08 ± 4.14 ml; *P* = 0.021). In the two groups, the numbers of unilateral lymph nodes harvested were similar. The differences in the postoperative hospital stay, skin, and lymphatic complications were not statistically significant.

**Conclusion:**

Compared with VEIL-H, gasless single-port laparoscopic inguinal lymphadenectomy through vulva incision reduces the difficulty of operation with shorter operation time, and less blood loss, which can be a safe and mini-invasive surgical approach.

## Introduction

Vulvar cancer is the fourth most common gynecological malignancy, which accounts for 5% of all gynecological malignant tumors (1). According to the estimation in 2020, 6,120 women were diagnosed with vulvar cancer and 1,350 women will die from it in the United States (2). The treatment of vulvar cancer is mainly surgery, supplemented by radiotherapy and chemotherapy. Lymphatic metastasis is the main transfer approach. Therefore, inguinal lymph node resection is a significant part of radical vulvectomy (RV) for vulvar cancer (3–5). As a traditional radical surgery for vulvar cancer, extensive resection of vulva + open inguinal lymphadenectomy (OIL) has a good tumor reduction effect. However, the life quality of patients was extremely affected by postoperative complications, such as inguinal incision infection, incision dehiscence, incision scar contracture, lymphocele, and lymphedema. With the continuous update and development of minimally invasive technology, laparoscopic technology has been widely applied in the field of surgery. VEIL in vulvar cancer has also increased. VEIL-H and the limb subcutaneous surgical approach (VEIL-L) are included. It has been confirmed that VEIL can significantly reduce postoperative complications, including wound infection, poor healing, and lymphedema. However, VEIL has difficulty in ' Bridge area ' lymph node dissection between the vulva and inguinal region and has the risk of tumor recurrence (6, 7).

Furthermore, a 6 cases series report single site lymphadenectomy through bilateral additional inguinal incisions for vulvar or vaginal cancer was safe (8). However, The risk of incisional complications and pneumo-related complications are still increased by another two incisions. Laparoendoscopic single-site surgery (LESS), or single incision laparoscopy, refers to the use of a single small skin incision to complete laparoscopic surgery. Recent advances in instrumentation, especially the single-site robotic platform, LESS is becoming more widely used in the field of minimally-invasive robotic and laparoscopic surgery in gynecologic oncology (9, 10). In this study, a gasless single incision through the vulvar endoscopic inguinal lymph node dissection was innovated for the first time. We retrospectively compared the clinical data of VEIL-H and VEIL-V and evaluated the technical points, feasibility, and efficacy of VEIL-V.

## Materials and Methods

### General Data

The clinical data of 22 patients, diagnosed as vulvar squamous cell carcinoma by histopathology, from January 2018 to March 2021 were retrospectively analyzed. In 13 cases before January 2020, the operation method was VEIL-H; in 9 patients after January 2020, the operation method was VEIL-V. All patients have excluded lymph node enlargement through pelvic MRI examination. Treatment includes unilateral or bilateral inguinal lymph node dissection, wide local excision, and radical Vulvectomy. The same surgical team performed all operations. The clinical data of the two groups are listed in Table 1, including age, BMI, Tumor diameter, Growth type (lateral, midline) and pathological staging. This study was approved by the Ethics Committee of the First Affiliated Hospital of Wannan Medical College [2020(04)]. All patients provided informed consent before surgeries.

**Table 1:** General data of patients.

**Clinical features**	**VEIL-V**	**VEIL-H**	***P***
Age (years)	65.33 ± 4.92	69.62 ± 2.33	0.396
BMI (kg/m^2^)	23.11 ± 0.67	23.62 ± 1.17	0.745
Tumor diameter	3.67 ± 0.91	2.92 ± 0.40	0.415
Growth type			0.609
lateral	3	2	
midline	6	11	
FIGO stage (2009)			0.962
I	7	10	
II–IV	2	3	

### Preoperative Preparation

Preoperative preparation: (a) Preoperative control of basic diseases, lower extremity arterial and venous ultrasound to understand the lower limb blood supply and exclude thrombosis, ureteroscopy, and anal finger examination to exclude adjacent organ metastasis; (b) The vulva and vagina were rinsed twice a day for two days with chlorhexidine before the operation. Intestinal preparation, fasting, and water deprivation for 8 h before the operation and enema once. Preoperative vulva skin preparation; (c) Anesthesia: intravenous combined anesthesia through endotracheal intubation.

### Surgical Steps

#### VEIL-H Surgery

Mark the surgical area. The triangle area formed by pubic tubercle, the anterior superior iliac spine, and 3 cm below femoral artery pulse position is the scope of lymph node dissection.

- Make a incision with a diameter of 1.0 cm at the lower edge of the umbilicus. The subcutaneous fat space was separated to the groin area. Laparoscopy lens implantation after CO_2_ cavity formation. Make two 10 mm and 5 mm incisions respectively, as the operating holes at the midpoint between the umbilical cord and the pubic symphysis as well as between the umbilical cord and the right anterior superior spinous cord.
- Seperate the fat and lymph nodes from the superficial fascia. The anatomical area is upward to the inguinal ligament, downward to the tip of the triangle, outward to the anterior superior iliac spine, and then inward to the internal pubic tubercle. Ultrasonic scalpel dissects and removes inguinal lymph nodes sequentially.
- Insert the drainage bag into the right incision. Suture skin puncture hole with 3–0 Vicryl.
- Radical excision of vulva according to the location and size of the lesion.

#### VEIL-V Surgery

Mark the surgical area as above.

**Figure 1:**
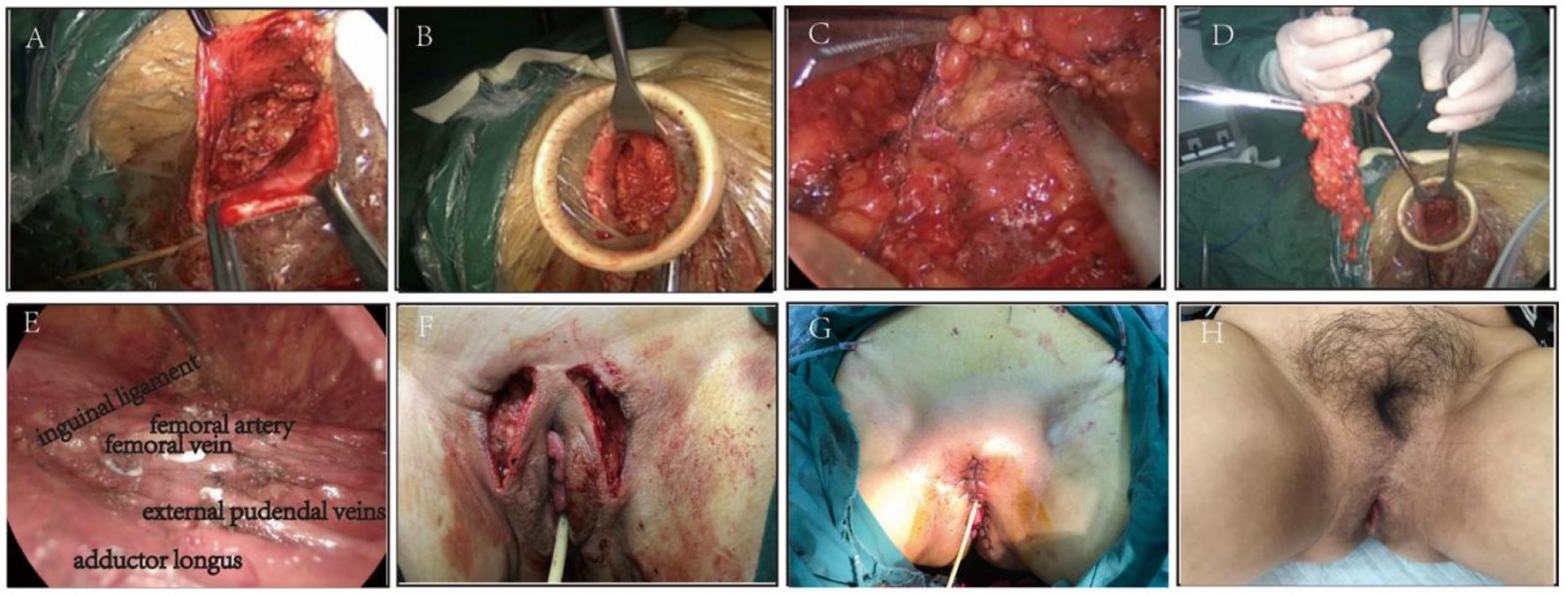
**(A)** Make a single incision at the outer margin of the lesion. **(B)** Create the surgical field with a suspension hook. **(C,D)**. Cut off the superficial and deep inguinal lymph node under the endoscope (Right side). **(E)** Complete removal of lymph nodes (Left side). **(F)** Performance after lymph node removal. **(G)** Performance after radical vulvectomy. **(H)** The appearance of the vulva six months after the operation.

- Mark the scope of vulvar resection according to the location and size of vulvar lesions. Make a single incision about 3 cm at the outer margin of the lesion, and separate the subcutaneous fat gap until entering the inguinal region. Create the surgical field with a suspension hook and wound retractor.
- Ultrasonic scalpel (Harmonic, Johnson & Johnson, California) outer upper and outer lower in two directions, fan-shaped to expand the separation of the subcutaneous fat gap, up to the medial anterior superior iliac spine, down to the vertex of the femoral triangle.
- Raise the lymphatic fat pad in the femoral triangle area, and separate and expose the inner and outer edges of the long adductor muscle with the ultrasonic scalpel.
- Under the endoscope, cut off the superficial and deep inguinal lymph node from the medial edge of adductor longus muscle to sartorius muscle, and protect the integrity of the great saphenous vein and accessory great saphenous vein. Cut off the superficial and deep inguinal lymph tissue in one piece at the inside of the fossa ovale and the root of the great saphenous vein.
- Radical excision of vulva and placement of subcutaneous drainage (Figure 1).

#### Estimated Bleeding Loss

Total bleeding volume (ml) = total blood gauze weight (g) – total dry gauze weight (g) + bleeding volume in suction bottle (ml), bleeding volume in suction bottle (ml) = total liquid volume in suction bottle (ml) – flush liquid volume (ml) during operation.

#### Postoperative Treatment

Prevent infection, and accurately record the volumes of the bilateral groins drainage fluid. Observed and recorded the postoperative complications, such as lower limb edema, embolism or thrombosis, lymphocele or lymphedema, and incision infection. All patients were followed up for 6 months after surgery.

### Statistical Analysis

All statistics were analyzed *via* the SPSS 23.0 (Inc., Chicago, IL, USA) software. Continuous data equasl to mean ± standard deviation (SD) through grouped comparison, which was performed by one-way analysis of variance (ANOVA). The χ^2^ test was used to assess Enumeration data with *P* < 0.05 indicated statistically significant. The distribution of data emerges on normal distribution (*P* > 0.05).

## Results

### Intraoperative Situation

In both groups, all operations were completed successfully. Three cases in groups VEIL-V and VEIL-H underwent unilateral lymphadenectomy, respectively, and all other cases underwent bilateral lymphadenectomy. VEIL-V had the advantages of shorter operation time and less blood loss than VEIL-H. There was no significant difference in the number of unilateral inguinal lymph nodes between the two groups (Table 2).

**Table 2:** Comparison of indexes during operation.

**Index**	**VEIL-V**	**VEIL-H**	***P***
operation time (min)	56.11 ± 5.94	74.62 ± 5.50	0.013
Number of unilateral lymph nodes	10.11 ± 1.05	9.92 ± 0.57	0.874
Blood loss (ml)	29.44 ± 2.56	43.08 ± 4.14	0.021

### Postoperative Short-Term Efficacy

Compared with the VEIL-H group, the drainage volume of the VEIL-V group was decreased. Skin complications occured in the inguinal region include skin infection, wound dehiscence, and skin necrosis. Lymphatic complications include lymphocyst, lymph secretion, and lymphedema. Follow-up for 6 months after surgery. In VEIL-V group, there was one case of wound dehiscence and secondary suture. One case of perineal incision infection and One case of lymphedema in VEIL-H group. There exist no significant difference in the postoperative hospital stay, skin and lymph complications in the inguinal region between the two groups after the operation (Table 3).

**Table 3:** Comparison of postoperative recent situation.

**Index**	**VEIL-V**	**VEIL-H**	***P***
Postoperative hospitalization (day)	9.89 ± 0.99	9.62 ± 1.04	0.857
Volume of drainage (ml)	125.6 ± 17.49	163.1 ± 25.55	0.029
Complications of groin skin	1	1	0.662
Lymphatic complications	0	2	0.338

## Discussion

Vulvar cancer is the fourth most common gynecological malignant tumors worldwide. North and South America, Europe, and Oceania are frequent-incidence areas, and Asia has the lowest incidence (11). There are 27,000 women diagnosed with vulvar cancer each year, more than 76 % of which is squamous cell carcinoma. Vulvar intraepithelial neoplasia is an important precursor. Surgery is considered as the standard treatment for vulvar cancer (12), including radical vulvectomy and radical inguino-femoral lymphadenectomy. While over the past two decades the radical nature of the procedure has changed or decreased. The complication rate of open inguinal lymph node dissection (OIL) can reach up to 70% (13). The postoperative complications include early lymphedema, seroma, necrosis, dehiscence, and wound infection, which are significantly correlated with the large inguinal incision required for adequate exposure. Furthermore, treatment can last for a long period and has a major difference to the quality of life (14, 15).

In 2010, Delman et al. first reported VEIL in patients with melanoma (16). VEIL includes the hypogastric subcutaneous approach and the limb subcutaneous surgical approach. Compared to the OIL, VEIL is effective and has more advantages, such as less bleeding, short hospitalization stay, and reduced postoperative complications (17). However, VEIL also has certain limitations, such as scars in the lower abdomen and difficult operation in case of burns in the inguinal region, great technical difficulty, and long surgical procedures (17, 18). More seriously, laparoscopy using CO_2_ can cause subcutaneous emphysema and even gas embolism (19).

Our VEIL-V group innovatively entered the inguinal lymph space from the exterior margin of vulvar resection without extra inguinal incisions and created surgical space by suspending the skin. The lymph nodes were removed under the microscope using the ultrasonic knife to avoid the subcutaneous tissue scar caused by the extra puncture hole, and it was easy to resect and remove the local lymph nodes. Compared with VEIL-H, it had a shorter operation time, less blood loss, and reduced operation difficulties. Furthermore, it is possible that the shorter VEIL-V operative time may actually be associated with improved surgical experience and skill. More importantly, the absence of CO_2_ not only avoids hypercapnia, subcutaneous emphysema, and gas embolism but also makes hemodynamics stable, which is suitable for elderly patients with heart, lung, and renal insufficiency. The perineal incision can be used to remove vulva lesions at the same time, without additional incision.

Compared to VEIL-H, it can be a safe surgical approach with more minimally invasion. For those with a larger scope of vulvar excision, the reconstructive techniques of the perineum after radical surgery, alongside VEIL-V, could increase the quality of life of these patients (20, 21). Since the choice of incision is located 2 cm from the outer edge of the tumor, this surgical method is not suitable for tumors close to the perineal union, which will cause inconvenience in operation.

Three modes of local recurrence were defined by Rouzier et al.: recurrence at the primary tumor site, distant recurrence (>2 cm to the primary tumor site), and skin bridge recurrence (6). Recurrence may occur in the dermis and the subcutaneous tissue between the groin and vulvar incision, which is described as skin bridge recurrence. Due to the problem of the approach angle, it is difficult for VEIL-H to clean the lymphatic tissue of the “bridge area,” so there is a risk of tumor recurrence. VEIL-V can dissect the ”bridge area” lymph fat tissue completely, theoretically reducing the recurrence rate of the tumor, which improves the postoperative survival rate of patients. A suspended gasless laparoscope can provide an ideal inguinal lymph node dissection field. After dissecting the lymph node, fingers can touch the surgical area, which reduces the possibility of local residual lymph nodes and improves the treatment outcomes.

Inguinofemoral lymph node dissection (ILND) has a wide range of operations. Postoperative complications, such as lymphedema (14–49%), lymphocyst (11–40%), and delayed wound healing are prone to occur (22), especially lymphedema of the lower extremities is one of the most severe long-term complications of ILND (23). To decrease morbidity and improve the testing of microscopic metastatic disease in the lymphatics, sentinel lymph node biopsy (SLNB) is widely utilized (24). Moreover, it was directional of lymphatic drainage from the vulvar tumor. The prime involved fields were superior-femoral fields and medial-inguinal. Therefore, sentinel lymph node biopsy through vulvar incision is more convenient, practical, and minimally invasive. Data from long-term follow-up research confirmed that in patients with early vulvar squamous cell carcinoma, SLNB did not show a higher recurrence rate or lower survival rate, and the tumor prognosis was not inferior to that of classic ILND (25). Due to the reduced complication rate found with the SLN technique in other types of gynecological cancers such as endometrial cancer (26), SLNB through vulvar incision maybe have good application prospects in the future in VEIL-V application.

The limitation of our research is the retrospective in nature. Small samples size is another limitation due to the incidence of diseases. This technique could not be applied in all vulvar cancer and need to have an appropriate patient selection. However, with the comparison group, we can validate the safety and efficacy of this procedure.

## Conclusions

To sum up, VEIL-V owns the characteristics of reaching the surgical field of VEIL-H, VEIL-V has obvious advantages in reducing the number of incisions, shortening the surgical approach, optimizing the surgical field, easing technical difficulty, reducing postoperative drainage volume, especially in the removal of the bridge area lymph node. However, due to the short follow-up as well as the small size of the sample, long-term efficacy is not yet known, which needs large prospective studies to clarify.

## Data Availability Statement

The raw data supporting the conclusions of this article will be made available by the authors, without undue reservation.

## Ethics Statement

The present study was approved by the Ethics Committee of the First Affiliated Hospital of Wannan Medical College [2020(4)]. The patients/participants provided their written informed consent to participate in this study.

## Author Contributions

GN and XG: conception and design of the research. JD and PT: wrote and revised the manuscript. JD: analysis and interpretation of the data and statistical analysis. SS, YL, HD, and XZ: provided all financial support, critical intellectual input in study design, and manuscript preparation. All authors contributed to the article and approved the submitted version.

## Funding

The open project of Key Laboratory of Non-coding RNA Transformation Research of Anhui Higher Education Institution (Wannan Medical College, RNA201901).

## Conflict of Interest

The authors declare that the research was conducted in the absence of any commercial or financial relationships that could be construed as a potential conflict of interest.

## Publisher's Note

All claims expressed in this article are solely those of the authors and do not necessarily represent those of their affiliated organizations, or those of the publisher, the editors and the reviewers. Any product that may be evaluated in this article, or claim that may be made by its manufacturer, is not guaranteed or endorsed by the publisher.
